# Psychotic-like experiences in non-clinical subgroups with and without specific beliefs

**DOI:** 10.1186/s12888-023-04876-9

**Published:** 2023-06-03

**Authors:** B. Hinterbuchinger, M. Koch, M. Trimmel, Z. Litvan, J. Baumgartner, E. L. Meyer, F. Friedrich, N. Mossaheb

**Affiliations:** 1 Department of Psychiatry and Psychotherapy, Clinical Division of Social Psychiatry, Medical University of Vienna, Waehringer Guertel 18-20, 1090 Vienna, Austria; 2 Section for Medical Statistics, Center for Medical Statistics, Informatics, and Intelligent Systems, Medical University of Vienna, 1090 Vienna, Austria

**Keywords:** Psychosis continuum, Psychotic-like experiences, Psychosis, Esoterism, Religion, Beliefs

## Abstract

**Background:**

Assuming a transdiagnostic and extended psychosis phenotype, psychotic-like experiences (PLEs) and psychotic symptoms are on a phenomenological and temporal continuum between clinical and non-clinical populations. Recent research points towards differences in PLE proneness in different subgroups and clinical impact of different PLE subtypes. This study examines the prevalence of PLEs in three groups of individuals with and without specific sets of beliefs aiming to elucidate the question whether proneness to PLEs varies according to traditional versus less traditional supernatural beliefs.

**Methods:**

The anonymized 16-item version of the Prodromal Questionnaire (PQ-16) was used to assess PLEs in three groups including individuals with religious beliefs (RB), belief in esoterism and paranormal phenomena (EB), and those embedded in scientific evidence approach and scepticism towards para-scientific theories (non-believers, NB). Male and female participants between 18 and 90 years were eligible for participation.

**Results:**

The sample comprised 159 individuals including 41 RB individuals, 43 EB individuals, and 75 NB individuals. The mean PQ-16 score of the EB individuals (6.86 ± 4.13) was significantly higher compared to NB individuals (3.43 ± 2.99) and to RB individuals (3.38 ± 3.23) with almost twice the score (both *p*-values < 0.001). There was no significant difference between the PQ-16 scores of the NB group and the RB group (*p* = 0.935). No significant impact of age (*p* = 0.330) and gender (*p* = 0.061) was found on the PQ16-Score.

Group affiliation to esoterism was associated with a higher PQ-16 score compared to group affiliation to religious beliefs (*p* < 0.001) and group affiliation to scepticism (*p* = 0.011), while the latter two did not differ significantly (*p* = 0.735). No significant difference was found between the three groups in the degree of distress related to the affirmatively answered PQ-16 items (*p* = 0.74).

**Conclusion:**

Under the assumption of a transdiagnostic psychosis phenotype, our findings provide more insight which subgroups within non-clinical samples have a higher likelihood of reporting PLEs.

## Significant outcomes

• Individuals with belief in esoterism self-reported significantly higher rates of psychotic-like experiences (PLEs) compared to individuals with religious beliefs and individuals with a scientific evidence approach and scepticism towards para-scientific theories.
• Individuals with religious beliefs and individuals with a scientific evidence approach had similar rates of self-reported PLEs.
• No significant difference in the degree of related distress was found between the three groups related to the affirmatively answered PQ-16 items.
• In-depth clinical interviews, phenomenological research on PLEs and longitudinal studies on mediating factors between PLEs and clinical impact within specific subgroups may clarify which individuals stay at the healthy end of the psychosis continuum and which develop psychiatric disorders.

## Limitations

• The use of self-report questionnaires developed for help-seeking individuals in non-clinical samples might result in an over- or underestimation of the prevalence of PLEs and psychotic symptoms in non-clinical samples.
• Since no in-depth clinical interview was performed, it is not possible to draw conclusions on whether individuals with belief in esoterism and with significantly higher rates of self-reported PLEs were reporting “true” PLEs or beliefs that are socio-culturally accepted within their specific subculture.
• The sample size of this study was relatively small and overlaps between beliefs cannot be explicitly ruled out within the compared groups.
• Due to the limited sample size, we could not correct for all confounding variables such as place of recruitment.

## Introduction

In contrast to the categorical approach of the “Kraepelinian dichotomy”, the concept of a transdiagnostic and extended psychosis phenotype assumes a temporal and phenomenological continuum [1, 2] from psychotic-like experiences (PLEs) in non-help-seeking individuals from the general population [3] to psychotic symptoms in individuals with non-psychotic psychiatric disorders such as anxiety disorders [4] as well as psychotic disorders including schizophrenia-spectrum-disorders [5]. The concept of psychosis as a transdiagnostic extended psychosis phenotype has resulted in the further examination of PLEs [6, 7] with a subsequently developing panoply of PLE terminologies, definitions and assessment tools [8]. Commonly used definitions of PLEs refer to “psychotic symptoms in the healthy general population”, “psychotic symptoms in the absence of illness” [7], or “psychotic symptoms in non-clinical populations” [3]. While these terms might remind of oxymorons, they do reflect the difficulties of using dichotomic notions within a conceptual continuum.

Diverging PLE assignments and assessment tools have resulted in mixed findings regarding prevalence and persistence rates and discussion about the clinical impact of PLEs [8]. Beyond being identified as early indicators of a later psychotic development with a fourfold increased psychosis risk in non-help-seeking individuals, PLEs also state non-specific markers for suicide risk, severe psychopathology impairments, depression, multi-morbidity and impairments of functioning [9–12].

While PLEs were found to be transitory phenomena in most cases, persistent psychotic experiences develop in 20%, with manifest psychotic disorders in 7% [10, 13–15]. Persistence of PLEs was found to be associated with genetic and environmental factors including trauma, substance abuse, levels of education and non-verbal IQ, urbanicity, as well as psychiatric comorbidities including depression or anxiety disorders and interactions between PLEs themselves [15–21].

PLE sub-categorization allowed for the assessment of differences concerning associated distress, need for treatment, comorbidities, functioning and psychosis risk with PLE subdimensions [1, 22–24]. The level of associated distress, a predictor of later need for treatment and the onset of manifest psychotic disorders [25, 26], as well as poor functioning and comorbid symptoms were found to vary widely between different PLE subtypes [6, 24, 27–29].

Besides more in-depth phenomenological research on PLEs, further identification and examination of modulating factors of proneness to PLEs might help to clarify the inconclusive role of PLEs along the psychosis continuum. For this purpose, there has been increasing interest in assessing PLEs in non-clinical subgroups with specific interest, beliefs or experiences in paranormal phenomena and esoterism [30, 31] which seem phenotypically similar to PLEs [32].

Paranormal phenomena are defined as “physically impossible” processes only explainable through revision of scientific fundamentals [33, 34], such as telepathy, clairvoyance, exorcism, telekinesis, mesmerism, reincarnation speaking in tongues, states of trance, possession, and several more; the umbrella term for these phenomena being esoterism [35]. What is perceived as paranormal or occult, depends—to a certain extent—on different socio-cultural norms [35]. Beliefs in the paranormal are common and widespread phenomena in the general population [36]. Nevertheless, there is evidence of interactions between belief in esoterism and the occurrence of psychotic and psychotic-like phenomena along a spectrum of PLEs [32, 33, 37], schizotypal traits [38, 39] and schizophrenia-spectrum disorders [40, 41], with similarities in cognitive processes [37, 42]. As shown in our previous study, individuals with interest in esoterism and paranormal phenomena report PLEs significantly more often compared to individuals without interest in esoterism [30]. Deriving from these results, the question has arisen whether proneness to PLEs in individuals with interest in esoterism are specifically related to esoterism itself or, on a broader level, to any supernatural “belief framework” including more traditionally and socio-culturally wide held beliefs, such as religious beliefs. As a matter of fact, religion has been described as acting both a potential risk factor and a protective variable [43–45]. In a large, cross-national study examining the relationship between lifetime PLE prevalence rates and religious affiliations in 18 countries, no significant association between different religious affiliations and PLEs were found. However, within the religious subgroup, those with very strong religious beliefs showed an increased risk of experiencing lifetime PLEs, regardless of any other psychiatric disorders [46].

The study of subgroups with specific beliefs might help to clarify the mechanisms which influence the expression of the psychosis phenotype. As a consequence, we assessed PLEs in individuals with two different belief frameworks, i.e. esoterism versus religion and compared these to scientifically embedded individuals who were assumed to be critical towards para-scientific beliefs.

The aim of this study is to elucidate the question whether proneness to PLEs varies with different sets of beliefs or whether supernatural belief per se makes one more prone to PLEs. In line with our previous study [30], we hypothesized that individuals with belief or interest in esoterism have significantly higher rates of PLEs compared to groups without paranormal beliefs, especially those with scepsis towards para-scientific ideas. We also hypothesized that individuals with more socionormative, namely religious beliefs have PLE rates located between those with belief in esoterism and those scientifically embedded individuals sceptical towards para-scientific beliefs.

## Material and methods

### Sample

Male and female participants between 18 and 90 years were eligible for participation. There were no further criteria for eligibility since we aimed to avoid a selection bias that may be found in studies with narrower inclusion criteria and those performed in clinical settings.

The sample of this cross-sectional study is a non-help-seeking population comprising 159 individuals self-assigned to three different groups: (i) 41 individuals with religious beliefs (RB), namely Christianity, which is the main religious confession in Austria with about 70% of the population identifying as Catholic (ÖIF 2017); (ii) 43 individuals with belief in esoterism and paranormal phenomena (EB), and (iii) 75 individuals strongly embedded in a scientific evidence approach and scepsis towards para-scientific theories (non-believers, NB). The study sample included 62 (39.0%) male participants and 97 (61.0%) female participants with 13 (32%) male and 28 (68%) female RB individuals, 4 (9%) male and 39 (91%) female EB individuals and 45 (60%) and 30 (40%) NB individuals.

**Fig. 1:**
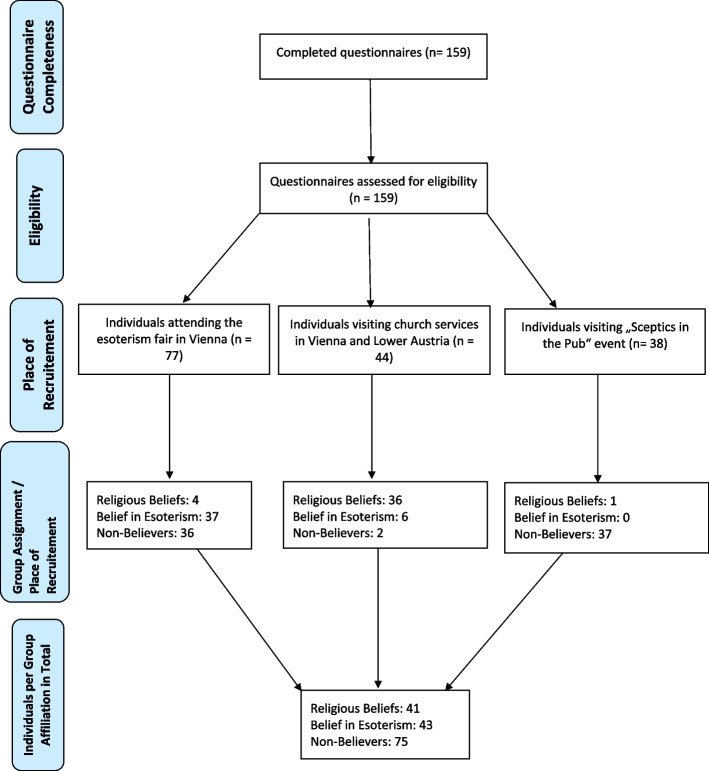
Depiction of the recruitment of the study participants according to group assignment

For the recruitment process see Fig. 1.

For sociodemographic statistics of the sample see Table 1.

**Table 1:** Sociodemographic statistics of age, gender, education and PQ-16 score w/r to belief/non-belief. Results for continuous variables (PQ16 score and Age) are presented as mean +—standard deviation, results for categorical variables (Gender and Education) are presented as absolute and relative frequencies (within belief groups)

		Group Affiliation			
		RB	EB	NB	*p*-value
PQ16_Score		3,38 +− 3,23	6,86 +− 4,13	3,43 +− 2,99	< 0.001
Age		49 +− 16	38 +− 12	38 +− 15	< 0.001
Gender	Male	13 (32%)	4 (9%)	45 (60%)	< 0.001
	Female	28 (68%)	39 (91%)	30 (40%)	< 0.001
Education	Compulsory	6 (15%)	7 (16%)	2 (3%)	*p* = 0.80
	A-levels/Apprenticeship	18 (44%)	24 (56%)	22 (29%)	< 0.001
	University	17 (41%)	12 (28%)	51 (68%)	< 0.001

*RB* individuals with religious beliefs, *EB* individuals with belief in esoterism and paranormal phenomena, *NB* non-believers

### Procedure

The purpose of the study was addressed in a short, written information at the beginning of the questionnaire. Group affiliation was assessed through one multiple-choice-forced-choice question. Participants were asked to self-rate to which of the three mentioned groups (belief in esoterism/religious beliefs/scientific approach with scepsis towards para-scientific beliefs) they felt most strongly affiliated to. Prior to the actual recruitment, this question was circulated to more than ten different non-clinical individuals to assure its intelligibility.

With respect to the three groups, recruitment was performed (i) at an esoterism fair at a large event venue in Vienna, Austria (https://www.esoterikmesse.de), (ii) after Catholic Sunday church service in two different churches in Austria (Vienna; Brunn am Gebirge in Lower Austria) and (iii) at events of the Austrian society for critical thinking which is part of the “Society for the scientific investigation of parasciences” (https://www.gwup.org) individuals strongly embedded in a scientific evidence approach and scepsis towards para-scientific theories such as esoterism. The esoteric fair was visited by approximately 1800 individuals within the fair’s duration of two days. Regarding the other recruitment places, approximately 120—150 individuals attended the church service and about 50 individuals visited the gathering of the “Society of critical thinking”. Recruitment of further study participants was performed via concentric-circle recruitment (e.g. other individuals regularly visiting the church were recruitment took place before church services). Individuals were asked whether they were attending the esoterism fair, church services or the scientific events and whether they were willing to anonymously participate in the study including the 16-items PQ and three additional questions on gender, age and group affiliation. No further information was given addressing the study. Participants were recruited between september 2019 and november 2019.

## Materials

### The 16-item version of the prodromal questionnaire (PQ-16)

Developed on the basis of the Prodromal Questionnaire (PQ) [47], the 16-item version of the PQ is a self-report screening questionnaire aiming to identify individuals with high risk for psychosis in help-seeking populations for a further in-depth diagnostic interview [48]. The presence of PLEs is assessed on a dichotomized scale (true/false) with 16 items including nine items assessing perceptual abnormalities and hallucinations, five items regarding unusual thought content, delusional ideas and paranoia and two items assessing negative symptoms. Associated distress was also assessed for each affirmed item (no distress, mild distress, moderate distress, severe distress). The total PQ-16 score is calculated from the number of items with response of “true” and ranges from 0 to 16 points. In help-seeking populations, correct classifications of high-risk states of psychosis or manifest clinical psychosis were found with a cut-off score of 6 or more positive items in the PQ-16 questionnaire in 44% of examined individuals differentiating those at ultra-high risk for psychosis (UHR) from individuals without UHR states with an equally high sensitivity and specificity of 87% [48]. Since our study examines PLEs in specific non-clinical samples with and without specific interests or beliefs with a screening questionnaire developed for help-seeking individuals, cut-off scores cannot be blindly taken into account within our sample. Albeit being developed for the use in help-seeking populations, there has been recent evidence suggesting that the PQ-16 states a valid instrument for assessing attenuated psychotic symptoms in non-help seeking populations [49]. An anonymized version of the questionnaire was used aiming to avoid social desirability biases and refusal to participate due to potential fear of stigmatization.

### Statistics

Descriptive statistics of variables of interest were compiled for each group separately. Sociodemographic variables were compared between the three different groups using ANOVA (continuous variables) and Chi-Square Tests (categorical variables). Mean PQ-16 scores of the three different groups were compared using ANOVA (overall) and t-tests (pair-wise). With one exception, all analyses in this paper compare the three groups RB, NB and EB with each other.

The comparisons of the NB group against the RB and EB group were considered the primary analyses in a pre-planned study protocol and Bonferroni multiplicity correction was applied (*p*-values < 0.025 were considered statistically significant). Furthermore, ANCOVA models were used to assess the influence of the group on the PQ-16 score, while correcting for the confounders age and gender and education (university degree versus no university degree). The influence of patient demographics on each single outcome of the PQ-16 questionnaire was investigated using logistic regression models (for these analyses we grouped patients by interest in esoterism, i.e. pooled patients from NB and RB to form a non-EB group). In order to investigate whether distress associated with affirmatively answered items of the PQ-16 questionnaire differed significantly between the three groups, a cumulative link mixed model was fitted accounting for the possible repeated measures of a single participant. For all secondary analyses, *p*-values < 0.05 were considered statistically significant as they serve only descriptive purposes. All analyses were conducted using IBM SPSS 25 and R 3.6.3. [50].

### Sample size calculation/power analysis

When the sample is 44 in each group, a two-sided t-test at significance level alpha = 0.025 has 80% power to detect a true difference in means of 0.667 standard deviations. Assuming a standard deviation of 3 points [30], this means a clinically relevant difference in means of more than 2 points can be detected with 80% power. We therefore aimed to include 44 patients per group.

## Results

The final sample comprised 159 individuals including 41 individuals with religious beliefs (RB), 43 individuals with belief in esoterism and paranormal phenomena (EB), and 75 non-believers/sceptics (non-believers, NB). Participants were between 18 and 78 years old. For sociodemographic statistics see Table 1.

### Main outcomes

#### Mean PQ-16 score

The mean PQ-16 score of the EB group (6.86 ± 4.13) was significantly higher compared to the NB group (3.43 ± 2.99) and compared to the RB group (3.38 ± 3.23) with almost twice the score (both *p*-values < 0.001). There was no significant difference between the PQ-16 scores of the NB group and the RB group (*p* = 0.935) (see Table 1).

### Secondary outcomes

#### Impact of age and gender on the PQ-16 score

Using an ANCOVA model, group affiliation showed a significant impact on the PQ-16 score (*p* < 0.001). Group affiliation to esoterism was associated with a higher PQ-16 score compared to group affiliation to religious beliefs (*p* < 0.001) and group affiliation to scepticism (*p* = 0.011), while the latter two did not differ significantly (*p* = 0.735). Age was not found to have a significant impact on the PQ16-Score (*p* = 0.330). Women had higher average PQ-16 scores than men (by 1.15 points), but the effect marginally missed statistical significance (*p* = 0.061).

Group affiliation to esoterism was associated with more affirmative answers compared to the two other groups, on 9 out of the 16 questions in the questionnaire (Question n.1, 4, 5, 6, 8, 9, 10, 13, 15; see Table 2). Significantly more women than men answered “yes” to question 2, 12 and 14 (see Table 2).

**Table 2:** Logistic regression for each PQ-16 item (Q1-Q16) w/r to age, gender and group affiliation esoterism; OR reflect chances to answer the question with “yes”, i.e. OR larger than 1 translate to increased odds of answering the question with “yes”, whereas OR smaller than 1 translate to decreased odds of answering the question with “yes”. Age refers to a one unit (i.e. year) increase in age

PQ 16 Items	Age	Sex Female	Group Affiliation Esoterism
1. I feel uninterested in the things I used to enjoy	OR = 0.98; *p* = 0.216	OR = 0.7; *p* = 0.442	OR = 2.72; *p* = 0.034
2. I often seem to live through events exactly as they happened before (déjà vu)	OR = 1; *p* = 0.772	OR = 2.74; *p* = 0.012	OR = 1.63; *p* = 0.221
3. I sometimes smell or taste things that other people can’t smell or taste	OR = 1.02; *p* = 0.121	OR = 1.95; *p* = 0.088	OR = 1.58; *p* = 0.257
4. I often hear unusual sounds like banging, clicking, hissing, clapping or ringing in my ears	OR = 0.98; *p* = 0.221	OR = 1.77; *p* = 0.272	OR = 4.34; *p* = 0.001
5. I have been confused at times whether something I experienced was real or imaginary	OR = 0.96; *p* = 0.017	OR = 1.82; *p* = 0.203	OR = 2.76; *p* = 0.021
6. When I look at a person, or look at myself in a mirror, I have seen the face change right before my eyes	OR = 1; *p* = 0.899	OR = 0.7; *p* = 0.547	OR = 7.34; *p* = 0
7. I get extremely anxious when meeting people for the first time	OR = 0.92; *p* = 0.073	OR = 3.62; *p* = 0.275	OR = 2.24; *p* = 0.347
8. I have seen things that other people apparently can't see	OR = 1.01; *p* = 0.534	OR = 1.32; *p* = 0.55	OR = 4.52; *p* = 0.001
9. My thoughts are sometimes so strong that I can almost hear them	OR = 0.98; *p* = 0.111	OR = 1.43; *p* = 0.42	OR = 2.44; *p* = 0.038
10. I sometimes see special meanings in advertisements, shop windows, or in the way things are arranged around me	OR = 1.01; *p* = 0.239	OR = 0.75; *p* = 0.452	OR = 3.67; *p* = 0.002
11. Sometimes I have felt that I’m not in control of my own ideas or thoughts	OR = 0.96; *p* = 0.012	OR = 0.94; *p* = 0.892	OR = 1.92; *p* = 0.139
12. Sometimes I feel suddenly distracted by distant sounds that I am not normally aware of	OR = 0.98; *p* = 0.053	OR = 2.46; *p* = 0.023	OR = 0.81; *p* = 0.61
13. I have heard things other people can't hear like voices of people whispering or talking	OR = 0.99; *p* = 0.408	OR = 2.13; *p* = 0.192	OR = 2.7; *p* = 0.044
14. I often feel that others have it in for me	OR = 0.99; *p* = 0.454	OR = 2.67; *p* = 0.025	OR = 1.56; *p* = 0.28
15. I have had the sense that some person or force is around me, even though I could not see anyone	OR = 1.02; *p* = 0.168	OR = 2.21; *p* = 0.052	OR = 5.12; *p* = 0
16. I feel that parts of my body have changed in some way, or that parts of my body are working differently than before	OR = 1.01; *p* = 0.372	OR = 1.27; *p* = 0.539	OR = 1.91; *p* = 0.115

*OR* odd's ratio

**Fig. 2:**
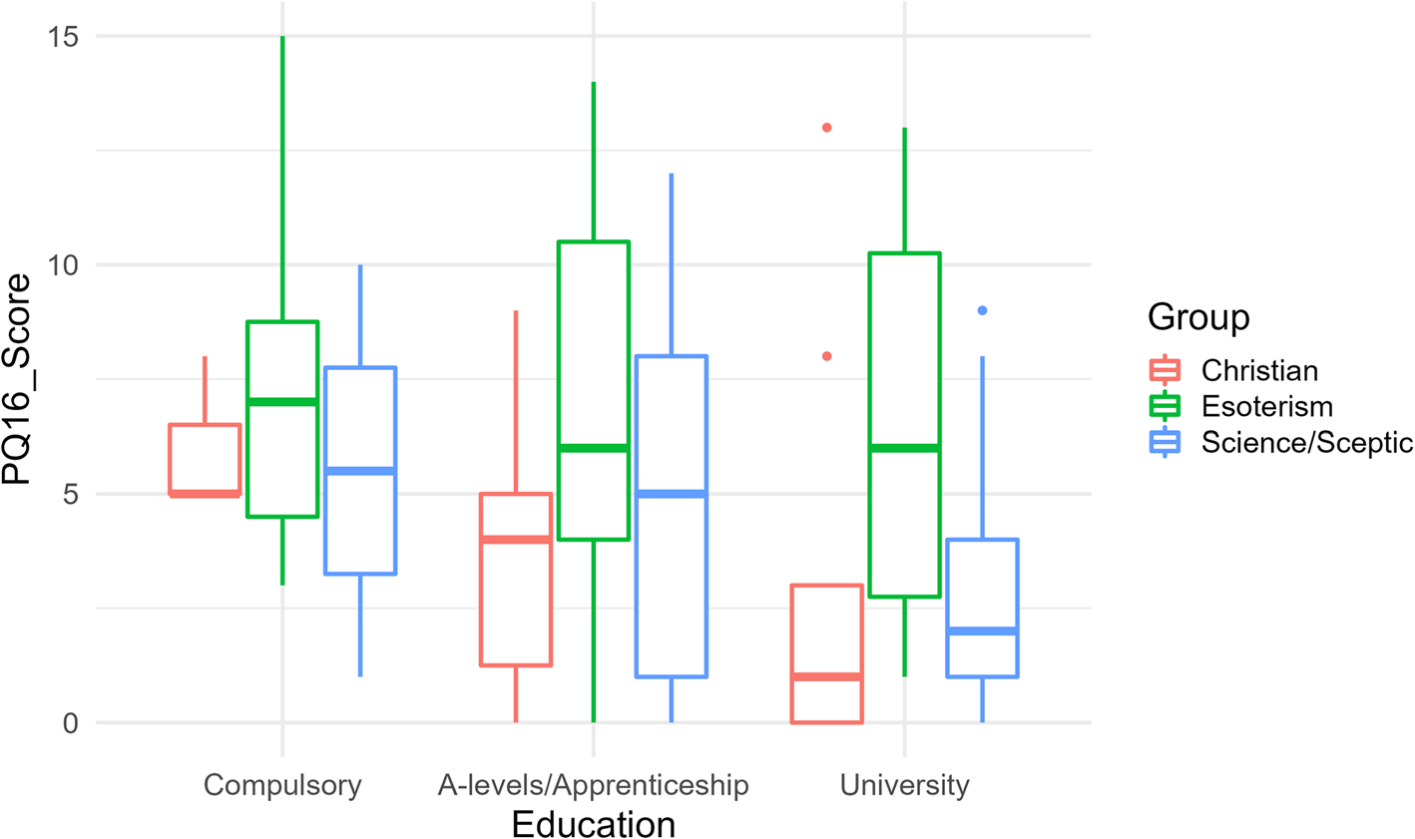
Boxplots of PQ-16 score w/r to education and group affiliation

Mean PQ-16 scores decreased with increasing education in all three groups. Within the two highest levels of education (University; A—levels/Apprenticeship), individuals with belief in esoterism showed significant differences in the mean PQ-16 mean scores compared to religious individuals and those skeptical towards para-scientific (both *p* = 0.001) with highest PQ-16 mean scores in individuals with belief in esoterism (see Fig. 2). Only within the level of compulsory education, there was no significant difference in the mean PQ-16 score between groups (*p* = 0.80). There was no significant difference between the three belief groups in the degree of distress associated to affirmatively answered items of the PQ-16 questionnaire (*p* = 0.74). University graduates had significantly lower average PQ-16 scores (by 1.70 points) than those without a university degree (*p* = 0.004).

## Discussion

In our study, individuals with belief in esoterism and paranormal phenomena had significantly higher scores on an anonymized self-report questionnaire assessing PLEs, compared to individuals with religious beliefs and to individuals with a scientific evidence approach and scepticism towards para-scientific theories. Secondly, albeit the fact that both religious and paranormal beliefs are defined as incorporating supernatural content outside the scope of scientific knowledge, the Christian RB participants of this study had mean PQ-16 scores almost identical to those of the NB group.

Thus, contrary to a supernatural belief hypothesis nurturing the presence of PLEs, religious and sceptic individuals in this study self-reported similar scores regarding PLEs, whereas esoterism aficionados reported on average twice as many PLEs. Significant sociodemographic differences were found between the three groups according to gender, age and education. However, age had no significant impact on the mean PQ-16 score and gender marginally missed statistical significance (*p* = 0.058).

### Paranormal beliefs—a non-clinical psychosis phenotype or coping mechanism?

The higher prevalence of self-reported PLEs in this sample of individuals with specific beliefs comes with the need to discuss possible interpretations of these results: Referring to a widely used definition, PLEs are “psychotic symptoms in the absence of illness” [7]. Thus, our data might indicate that individuals who believe in esoterism and experience PLEs without any associated distress, might be referred to as “healthy schizotypes” [39] or “non-clinical/subclinical psychosis phenotypes”, possibly sharing socio-environmental, psychopathological, developmental and genetic risk factors [1, 7] with schizophrenia-spectrum disorders without any associated distress. In contrast to other studies [24, 26, 27], we found no significant difference in the degree of distress associated with affirmatively answered items of the PQ-16 questionnaire between the three groups. As a matter of fact, the level of distress might be a significant parameter when interpreting PLEs. In fact, the level of distress has been shown to predict later need for treatment and the onset of manifest psychotic disorders [25, 26]. Also, previous studies found different PLE subtypes being associated with variations of associated distress and consequently the need for treatment, level of functioning, comorbidities and psychosis risk [1, 22, 24, 26]. Differences have further been reported with respect to PLE subdimensions [24, 27, 28]: While paranoid, hallucinatory and delusional PLEs were shown to strongly correlate with distress, paranormal beliefs and grandiosity were not found to be associated with distress [24]. Furthermore, paranormal beliefs and magical ideation were even inversely correlated with other psychiatric symptoms such as physical anhedonia [29] and depression [26]. Magical thinking, including similar or overlapping features such as paranormal beliefs, showed only a weak association with distress [24, 26].

It was previously hypothesized that for some individuals, paranormal beliefs might state a coping strategy and a response to stressful life events helping to maintain mental functioning by offering an “illusion of control” [51–53]. In line with this hypothesis, a history of trauma or adverse life events was found to be associated with higher levels of paranormal beliefs and PLEs [54–56]. However, in our study, no significant difference in the degree of related distress was found between the three groups related to self-reported PLEs.

Assuming that the subgroup with paranormal beliefs and higher rates of PLEs in our sample stays at the healthy end of the psychosis continuum, it is to question if perceptional aberrations and delusion-like ideas per se are related to any clinical impact or distress or if a number of individuals may experience psychotic-like phenomena without having or ever developing a psychiatric disorder. Resilience factors and cognitive impairments could state mediating factors between schizotypal personality traits or perceptual aberrations and the level of associated distress [57, 58]. Further examination of modulating factors between psychotic phenomena and distress within specific subgroups, as shown by Powers et al., [31], might contribute to a better understanding of psychotic phenomena and help to clarify the inconclusive role of PLEs along the psychosis continuum.

### Are paranormal beliefs indicators of mental disorders?

Another possible interpretation of our results would be that the subgroup of individuals with belief in esoterism who reported significantly more PLEs did actually experience manifest psychotic symptoms with a potential need-for-treatment or had a manifest psychotic disorder. First of all, we did not use the PQ-16 aiming to screen or assess psychotic symptoms and disorders, nor did we proceed to a psychiatric examination. Given the fact, that PLEs show highest prevalence rates at young age, e.g. in children and adolescents [13] with a decrease in PLE occurrence with higher age and that individuals in our sample were in their late adulthood, it cannot be ruled out that some individuals who self-reported higher PQ-16 scores in our sample might in fact have reported manifest psychotic symptoms. Support, explanations or help could possibly be sought within a specific subculture in which perceptual aberrations or idiosyncratic beliefs are more acceptable, even desired or interpreted in a specific way (e.g. thought intrusion as telepathy).

When continuing on progression along the psychosis continuum, we need to address clinical high-risk states for psychosis situated somewhere between non-clinical psychosis phenotypes and manifest psychotic disorders [59]. Clinical high-risk states may also be a possible explanation for significantly higher rates of PLEs in specific subgroups. Since clinical high-risk states are typically phenomena of individuals in their adolescence and early adulthood [60] and individuals in our sample were in their late adulthood, it does not seem very likely that higher scores of self-reported PLEs in EB individuals within our sample are related to clinical high-risk states of psychosis. Having said this, we cannot exclude the possibility, that some individuals with higher self-reported PLEs in the PQ-16 might in fact have experienced or self-reported manifest psychotic symptoms within our sample. Furthermore, the PQ-16 questionnaire is not sensitive enough to distinguish between psychotic symptoms and attenuated psychotic symptoms, such as in clinical high risk states [48]. However, while it is possible that individuals at ultra-high risk of psychosis or with manifest psychotic disorders are included in our study sample, we did not assess if or to which extent individuals in our sample have manifest psychotic or high-risk symptoms or psychotic disorders. We examined a non-clinical, non-help-seeking sample in a non-clinical setting, where people pursued their interests in their free time. The aim of this study was to examine if differences in self-reported PLEs in the PQ-16 assessment exist between individuals with specific (religious; esoteric) beliefs and those without specific beliefs (sceptics).

If PLEs are assumed as a transdiagnostic non-specific marker of impairment and psychiatric—not specifically psychotic—symptoms [1], the question emerges whether our results of higher self-reported PLE rates in individuals with belief in esoterism point towards an increased risk or higher rates of—not necessarily psychotic—psychiatric disorders, symptoms or psychological problems. Examining this question in non-clinical samples [61–63] has resulted in ambiguous findings: While no positive association was found between measures of clinical impact, including psychoticism and neuroticism, and paranormal experiences in a non-clinical sample of students [62], measures of fantasy proneness including paranormal experiences was strongly connected to neuroticism in another study sample of Spanish students [61]. Adolescents with paranormal beliefs about contacting dead people showed significantly higher rates of anxiety and worry, depressive symptoms, isolation and suicide thoughts compared to adolescents without these specific beliefs [63]. In clinical samples, paranormal beliefs—as well as religious beliefs—were found to be related to symptoms of obsessive–compulsive disorder [64]. However, within a more detailed exploration of the affirmatively answered items of the PQ-16 questionnaire in our study, EB individuals had a significantly higher risk of a positive answer compared to the other groups especially concerning items addressing the positive symptom dimension including hearing, feeling or seeing things that cannot be heard or seen by others, pointing towards the occurrence of hallucinations, hallucinatory experiences or illusions and seeing special meanings in the surroundings, indicating ideas of reference.

Our study design does not allow us to draw conclusions on the occurrence of manifest psychiatric disorders in our sample. Further studies with additional in-depth psychiatric assessments with larger sample sizes might be able to shed more light onto this question.

### *“It is not what you believe, it is how you believe it.”* [65] – is it really?

Albeit the fact that both religious and esoteric beliefs are defined as incorporating supernatural content outside the scope of scientific knowledge, RB individuals identifying with the most frequent religious confession in Austria, Christianity, showed no significant difference regarding self-reported PLE rates and mean PQ-16 scores almost identical to those of the NB group, thus varying widely from EB individuals relating to PLEs.

One explanation might be that certain, e.g. religious, beliefs become more and more normalized when shared within a large community over a long time period [66]. Another hypothesis indicates that originally psychotic-like ideations included within a religious framework of beliefs—e.g. the burning bush appointing Moses to lead the Israelites out of Egypt and into Canaan [67]—drift out of the populations’ focus during time and, within an abstracting procedure, develop into socio-culturally accepted and integrated traditions and rituals. Indeed, delusional ideations were found to be at a continuum between different belief frameworks: Religious groups with more unconventional beliefs such as Hare Krishnas and Druids extended further along the psychosis spectrum compared to more conventional religious and non-religious groups [65]. Variations in PLE proneness according to different sets of beliefs may point towards differences along a spectrum with potentially, but not necessarily, clinical relevance leading to the question which PLEs are on the psychosis continuum and have clinical impact and which are phenomena experienced by some individuals without distress or need for treatment.

Since previously paranormal beliefs were shown to be related to the degree of education with higher education being associated with less paranormal beliefs [68], the level of education was also assessed within this sample. Although in our data the mean PQ-16 score decreased with increasing level of education, significant differences between belief groups were found within two of three educational levels with highest mean PQ-16 scores in EB individuals compared to RB and NB individuals. Thus, we assume that education might not be the main factor relevant for proneness to PLEs or paranormal beliefs.

## Limitations

The use of a self-reporting PLE questionnaire developed for help-seeking individuals in a non-clinical sample can be seen as a limitation due to possible “false positive” answers self-reporting PLEs not confirmed in clinical interviews [69]. Some groups have argued that self-rating PLE assessment tools can result in an overestimation of the prevalence of clinician-rated psychotic symptoms and cannot be considered a valid approximation of attenuated psychotic symptoms [70–72]. However, we did not aim to detect or screen for manifest psychotic symptoms or individuals at risk of developing a psychotic disorder in our study but specifically aimed to address self-reported PLEs in specific subgroups with different set of beliefs. Moreover, although being criticized for having a sensitivity too low for recommending their use within a general screening for potential clinical high risk for psychosis [71], self-reported psychotic experiences in epidemiological non-help seeking samples were found to index risk for the development of later psychotic disorders [10]. Even “false positive”, e.g. not clinically validated, self-reported psychotic experiences were associated with the later development of psychotic disorders [73], clinically relevant psychotic symptoms, mood and anxiety disorders and reduced functioning [69] and were consequently discussed to be the softest expression of an extended psychosis phenotype within the psychosis continuum [74]. While PLE prevalence and incidence rates were found to be two–three times higher in self-report assessment instruments compared to clinical interviews, results on associated factors including risk factors were similar [20].

Future studies are needed to clarify whether higher PLE scores in individuals with specific sets of beliefs are associated with any clinical impact and if so, whether they indicate an association of PLEs with mental problems and disorders in general or are specifically related to potentially pre-psychotic or psychotic conditions [75, 76]. To answer the question about the possible clinical impact of the self-reported PLEs in this study, in-depth clinical interviews with standardized instruments and psychiatric examinations would be necessary in future studies. Our study was designed to assess PLEs with an anonymized version of the questionnaire in this sample to avoid major social desirability biases, as well as refusal to participate due to fear of stigmatization.

Furthermore, our sample size was relatively small. Studies with larger sample sizes might be necessary to replicate our findings and also further assess the role of possible variables such as age and gender in larger groups as well as any potential association to psychiatric comorbidities, vulnerability or other psychiatric symptoms. Overlaps between beliefs cannot be explicitly ruled out within the compared belief groups, however, participants had to select a multiple-choice-forced-choice question to self-rate to which of the three mentioned belief groups they feel most strongly affiliated to. Furthermore, due to the limited sample size, we could not correct for further confounding variables such as education or place of recruitment.

Psychotic symptoms are characterized as being idiosyncratic, not shared by other individuals, and out of touch with the dominant subculture [77]. This raises the question how “true PLEs” would phenomenologically appear in a sample of individuals with shared beliefs, such as paranormal beliefs. Since in this study no in-depth clinical interview was performed, it is not possible to draw conclusions on whether individuals with belief or interest in esoterism and with significantly higher rates of self-reported PLEs were reporting “true” PLEs or psychotic symptoms or beliefs and ideas that are socio-culturally accepted within their specific subculture. Further, in a sample of individuals with shared idiosyncratic beliefs, specific experiences might even be desired with the potential of increasing the probability of answers in the affirmative. We also did not assess the “degree” of the participants’ beliefs, thus, we cannot answer the question regarding variations concerning the degree of the personal conviction associated with specific beliefs.

Since the PQ-16 was developed as a screening instrument for help-seeking populations in secondary mental health care services [48], the instrument´s sensitivity could be too high when examining a sample of shared idiosyncratic beliefs, that phenomenologically similar to PLEs and psychotic symptoms.

Another limitation of this study might be the fact that individuals recruited at specific locations were in most cases—but not always—identifying themselves with the “assumed” set of belief, for example did not all individuals recruited at church services declare themselves as “most strongly embedded in religious beliefs” but also as “non-believers” or “believers in esoterism”. Statistical analyses concerning differences in PQ-16 scores were performed between the self-declared set of belief and we did not control for a potential confounding bias resulting from place of recruitment. Moreover, the results of our study cannot be generalized to the general population, since we analyzed small samples of specific subgroups, i.e. only Christians in the subgroup of religious believers and subgroups which might also represent specific variations of their (non-) beliefs with engagement in specific organizations, such as the “Society for critical thinking”.

Due to the very busy and highly frequented recruitment settings with limited time ressources of the visitors e.g. at public events such as the esoterism trade or before church services, it was not feasible to record how many individuals who were verbally addressed and asked to participate declined study participation during recruitment. However, due to our small sample size, we are aware that our three samples are not representative for the, as we assume, very heterogenous groups of individuals with religious beliefs, individuals with belief in esoterism and those individuals sceptical towards non-scientific theories prone to an evidence-based scientific approach.

## Conclusion

Our results show significant differences in self-reported PLE between different groups of individuals with specific beliefs with higher rates in individuals with belief in esoterism compared to individuals with religious beliefs and scepticism towards paranormal phenomena. Aiming to examine the mechanisms which influence PLE proneness, our findings suggest that proneness to PLEs in people with interest in esoterism is specifically related to group affiliation to esoterism itself and not, on a broader level, to any supernatural „belief framework” including more traditionally and socio-culturally wide held beliefs, such as religious beliefs.

In contrast to previous studies reporting less or no distress associated with specific PLE subtypes such as paranormal beliefs and magical thinking [24, 26, 27], no significant difference in the degree of related distress was found between the three groups related to self-reported PLEs.

Examination of PLEs in different subgroups might help to understand which individuals might have a higher likelihood for experiencing PLEs. For a better understanding of the clinical relevance of PLEs in specific subgroups, further longitudinal studies [78] on mediating factors between PLEs and clinical relevance along the psychosis spectrum [57], as well as more phenomenological research on PLEs in subgroups with high prevalence rates of PLEs seem necessary.

## Data Availability

The datasets used and/or analysed during the current study are available from the corresponding author on reasonable request.
