# Correction to: Psychotic-like experiences in non-clinical subgroups with and without specific beliefs

**DOI:** 10.1186/s12888-023-04968-6

**Published:** 2023-07-10

**Authors:** B. Hinterbuchinger, M. Koch, M. Trimmel, Z. Litvan, J. Baumgartner, E. L. Meyer, F. Friedrich, N. Mossaheb

**Affiliations:** 1 Department of Psychiatry and Psychotherapy, Clinical Division of Social Psychiatry, Medical University of Vienna, Waehringer Guertel 18-20, Vienna, 1090 Austria; 2 Section for Medical Statistics, Center for Medical Statistics, Informatics, and Intelligent Systems, Medical University of Vienna, Vienna, 1090 Austria

BMC Psychiatry (2023) 23:397

10.1186/s12888-023-04876-9

In the original publication of this article the PDF did not contain the correct figures, these were correct in the web version. The original publication has been updated. The publisher apologizes for the inconvenience to the authors & readers.

